# Human Endogenous Retrovirus and Neuroinflammation in Chronic Inflammatory Demyelinating Polyradiculoneuropathy

**DOI:** 10.1016/j.ebiom.2016.03.001

**Published:** 2016-03-10

**Authors:** Raphaël Faucard, Alexandra Madeira, Nadège Gehin, François-Jérôme Authier, Petrica-Adrian Panaite, Catherine Lesage, Ingrid Burgelin, Mélanie Bertel, Corinne Bernard, François Curtin, Aloïs B. Lang, Andreas J. Steck, Hervé Perron, Thierry Kuntzer, Alain Créange

**Affiliations:** a GeNeuro Innovation, France; b Reference Center for Neuromuscular Diseases, Department of Pathology, Henri Mondor Hospital, Créteil, France; c INSERM U955-Team 10 Biology of the Neuromuscular System, Paris Est-Creteil University, Créteil, France; d Department of Clinical Neuroscience, Lausanne University Hospital (CHUV), Switzerland; e GeNeuro SA, (Geneva), Switzerland; f Department of Neurology, Henri Mondor Hospital, APHP, Université Paris Est, Créteil, France

**Keywords:** CIDP, Peripheral neuropathies, Schwann cell, Endogenous retrovirus, HERV, HERV-W, MSRV, GNbAC1

## Abstract

**Background:**

Human endogenous retroviruses HERV-W encode a pro-inflammatory protein, named MSRV-Env from its original identification in Multiple Sclerosis. Though not detected in various neurological controls, MSRV-Env was found in patients with chronic inflammatory demyelinating polyradiculoneuropathies (CIDPs). This study investigated the expression of MSRV in CIDP and evaluated relevant MSRV-Env pathogenic effects.

**Methods:**

50 CIDP patients, 19 other neurological controls (ONDs) and 65 healthy blood donors (HBDs) were recruited from two different countries. MSRV-env and -pol transcripts, IL6 and CXCL10 levels were quantified from blood samples. MSRV-Env immunohistology was performed in distal sensory nerves from CIDP and neurological controls biopsies. MSRV-Env pathogenic effects and mode of action were assayed in cultured primary human Schwann cells (HSCs).

**Findings:**

In both cohorts, MSRV-env and -pol transcripts, IL6 positivity prevalence and CXCL10 levels were significantly elevated in CIDP patients when compared to HBDs and ONDs (statistically significant in all comparisons). MSRV-Env protein was detected in Schwann cells in 5/7 CIDP biopsies. HSC exposed to or transfected with MSRV-env presented a strong increase of IL6 and CXCL10 transcripts and protein secretion. These pathogenic effects on HSC were inhibited by GNbAC1, a highly specific and neutralizing humanized monoclonal antibody targeting MSRV-Env.

**Interpretation:**

The present study showed that MSRV-Env may trigger the release of critical immune mediators proposed as instrumental factors involved in the pathophysiology of CIDP. Significant MSRV-Env expression was detected in a significant proportion of patients with CIDP, in which it may play a role according to its presently observed effects on Schwann cells along with previously known effects on immune cells.

Experimental results also suggest that a biomarker-driven therapeutic strategy targeting this protein with a neutralizing antibody such as GNbAC1 may offer new perspectives for treating CIDP patients with positive detection of MSRV-Env expression.

**Funding:**

Geneuro-Innovation, France.

## Highlights

• A protein from endogenous retroviruses (8% of human genome) is found in chronic inflammatory demyelinating polyradiculoneuropathies (CIDP).
• Identified in multiple sclerosis, MSRV-Env induces inflammation and autoimmunity.
• It is now shown in peripheral nerve lesions, serum and blood cells of CIDP patients.
• In Schwann cells, it induces pro-inflammatory factors; the same is detected in serum.
• Antibody neutralization of MSRV-Env effects raises therapeutic perspectives in CIDP.

Since the 1980's, a new horizon in the search for pathogenic players in complex human disease arose from studies in which human endogenous retrovirus families (HERV) were identified. HERVs represent 8% of the human genome and may express pathogenic proteins as shown here in a chronic inflammatory demyelinating polyradiculoneuropathies (CIDPs). The present study shows that MSRV-Env, an HERV protein, is detected in CIDP lesions and blood. It triggers inflammation in immune and nervous cells. A specific antibody revealed efficient in experimental conditions. This offers new therapeutic and diagnostic perspectives for this disabling disease without curative treatment.

## 1 Introduction

Human endogenous retroviruses (HERVs) originate from ancestral integrations of exogenous retroviruses during evolution and represent 8% of the human genome, in which most copies are inactivated or silenced (Belshaw et al., 2005). However, a retroviral element expressing proteins was isolated in Multiple Sclerosis (MSRV, for Multiple Sclerosis associated RetroViral element) and unveiled a family of homologous endogenous copies (HERV-W) (Blond et al., 1999, Perron et al., 1997, Perron et al., 1991). The HERV-W family comprises multiple copies inserted in the human genome. One of them has been domesticated throughout evolution and encodes an HERV-W envelope, named Syncytin (Mi et al., 2000) for its original fusogenic properties involved in the physiological development of the syncitio-trophoblast tissue in the placenta (Frendo et al., 2003). It is selectively expressed during placentation, is transcribed from a locus (ERVWE1) within a defective HERV-W copy on chromosome 7 and has a unique molecular signature among HERV-W envelope sequences (Bonnaud et al., 2004, Mallet et al., 2004). This protein and its coding nucleotide sequences can thus be differentiated from the envelope sequences obtained from genomic RNA in purified retroviral particles from MS (Mameli et al., 2009). The latter define an MSRV-subtype of HERV-W elements that comprises multiple related defective fixed copies in the human genome such as, e.g., a partial HERV-W copy on chromosome X that potentially encodes a truncated envelope (ERVWE2 locus) and may interfere with MSRV expression (Roebke et al., 2010, do Olival et al., 2013, Garcia-Montojo et al., 2014). HERVs are not infectious viruses but human DNA sequences related to retrotransposable genetic elements, few of which have the potential to be activated by various environmental triggers, including infectious viruses on a “hit-and-run” mode (Perron and Lang, 2010, Mameli et al., 2012). HERV-W proteins are tolerated by human adaptive immune system and neither antibody nor T-cell response to HERV-W proteins can be seen, unless in rare and extreme conditions that may relate to autoimmunity (Ruprecht et al., 2008).

Beyond this fundamental research context, independent studies confirmed an association of MSRV expression with MS (Perron et al., 2012, Sotgiu et al., 2010). Its envelope protein (MSRV-Env) was shown to elicit pro-inflammatory and autoimmune responses in immune cells (Perron et al., 2001, Rolland et al., 2006) and to impair remyelination by oligodendrocyte precursor cells (OPCs) (Kremer et al., 2013), suggesting its involvement in MS pathogenesis (Perron et al., 2012, Kremer et al., 2014, Madeira et al., 2016).

Chronic inflammatory demyelinating polyradiculoneuropathy (CIDP) is a rare immune disease of the peripheral nervous system (PNS), with multifocal inflammatory and demyelinating lesions in nerve roots also expanding to distal regions (Vallat et al., 2010). Its clinical presentation is heterogeneous and its diagnosis is challenging without known etiology or specific biomarkers (Dalakas, 2011, Anon., 2008, Anon., 2010, Koller et al., 2005). CIDP therapies are intravenous human immunoglobulins (IVIG), corticosteroids or plasma exchange. Long-term therapy is often limited by side effects and one-third of patients are refractory to existing treatments (Latov, 2014), which illustrates the unmet need for diagnostic biomarkers and innovative treatments of CIDP.

In a previous study on MS, MSRV-Env was not detected in healthy controls and in various other Neurological Diseases except for CIDP cases (5/8) (Perron et al., 2012). This observation prompted the present study to investigate a potential association of this endogenous HERV-W element with CIDP. Its results have confirmed significant MSRV expression in CIDP, have demonstrated the pathogenic effects of MSRV-Env on human Schwann cells (HSC) and their inhibition by GNbAC1, a neutralizing and highly specific humanized antibody targeting this MSRV-Env endogenous protein.

## 2 Patients and Methods

### 2.1 Origin of Samples

The overall study group consisted of 51 CIDP patients, from the Neurology Departments of Henri Mondor University Hospital (Creteil, France) and of Vaudois University Hospital (CHUV-Lausanne, Switzerland). CIDP patients fulfilled criteria of the EFNS and PNS Joint Task Force guidelines (PNS JTFOTEat, 2010). The majority of patients had symmetric sensorimotor deficits (24 patients), 9 patients had asymmetric sensorimotor deficits, MADSAM type, and one patient had pure sensory deficit. The number of patients in each category is too small to consider statistical interpretation on these symptomatologic subgroups. 19 patients with other Neurological diseases (OND) were recruited in Creteil Neurology department: type 2 diabetes associated neuropathies (n = 8), Parkinson disease (n = 3), diffuse myalgias without a known cause (n = 2), stroke, meningoradiculitis, macrophagic myofasciitis, axonal idiopathic neuropathy, Guillain–Barré syndrome, and spastic paraparesis (n = 1 each). 65 healthy blood donors (HBDS) from CHUV Lausanne or from Etablissement Français du Sang, Annemasse, France, provided samples. Written informed consent to use their blood for research analyses after anonymization was obtained from all individuals. The study protocol was approved by local research ethics committees (Créteil: ethics committees CPPIDF VI and CPPIDF IX, POLYCHROME study number ID RCB 2010-A01226–33; Lausanne: protocol 235/10).

In a first cohort (Study 1), 20 CIDP patients from Creteil and 21 HBDs from Annemasse, were enrolled. A second cohort (Study 2) included 11 additional CIDP patients and 19 OND patients from Créteil, 20 CIDP patients and 18 HBDs from Lausanne, and 26 HBDs from Annemasse, for a total of 31 CIDPs, 19 ONDs and 44 HBDs in study 2. MSRV transcript levels in PBMC were assessed separately in Studies 1 and 2. In the light of results obtained in human Schwann cells,

IL6 and CXCL10 serum levels were determined retrospectively, at the same time for both cohorts (aliquoted samples from studies 1 and 2).

For serum, 6 mL of blood was collected on a dry tube and 500 μL of serum aliquots were frozen at − 80 °C. For peripheral blood mononuclear cells (PBMCs), 4 mL of blood was collected in a Cell Preparation Tube (ref. 362781, Becton Dickinson, Paris, France) and treated according to the manufacturer's instructions. PBMCs in heat-inactivated Fetal Calf Serum with 10% dimethyl-sulfoxide were stored at − 80 °C.

### 2.2 Quantification of MSRV-env and -pol Transcripts in PBMC by Real-time RT-PCR (qRT-PCR)

Thawed PBMCs were washed with PBS (1700 g for 20 min at 10 °C). Total RNA was isolated with QIAamp RNeasy Mini Kit (Qiagen, Courtaboeuf, France) and treated with Turbo DNA-Free™ (Life Technologies, Saint-Aubin, France) according to the manufacturer's instructions. RNA concentration was assessed with a Nanodrop 2000 (Fisher Scientific, Illkirch, France) before adjustment to 10 ng/μL with RNase-free water. First-strand cDNA was synthesized with i-script select cDNA-synthesis kit using oligonucleotide dT (18) (BioRad, Marnes-La-Coquette, France) at 42 °C for 60 min, inactivated at 85 °C for 5 min and adjusted to 10 ng μL^− 1^ with RNase-free water. 50 ng of cDNA was used with iQ supermix (BioRad, Marnes-La-Coquette, France) and corresponding sets of primers/probes for qRT-PCR. The internal control was glucuronidase beta gene, GUS B (Taqman gene expression assay GUS B, Life Technologies, Saint-Aubin, France) and specific sets of primers and probes for MSRV-env as described (Mameli et al., 2009). MSRV-pol transcripts were quantified with a FAM™ fluorescent reporter (forward primer: 5′-CCTGTACGTCCTGACTCTC-3′; reverse primer: 5′-CTTGGGCTAATGCCTGGCC-3′; probe: FAM-CCAACGTCTCAACTCACCTGG-TAMRA). PCR was performed with a C1000 thermal cycler and a CFX96 real-time system (BioRad, Marnes-La-Coquette, France), with an initial denaturation step (95 °C, 10 min) followed by 45 cycles of successive denaturation (95 °C for 10 s) and annealing/extension (60 °C, 1 min) steps. For each sample, the expression of MSRV transcripts and GUS B was calculated as the cycling threshold (Ct), assessed in triplicates, and MSRV transcript level was expressed as relative expression to GUS B, according to the ΔCt method with reference gene (Real-Time Application Guide, BioRad, Marnes-La-Coquette, France). For each sample, a control without reverse transcriptase (No RT) was performed to detect eventual DNA contamination. Results validation required: negative No RT control, PCR efficiency between 90 and 110% with slope between 3.1 and 3.6, and triplicate variation below 5%. In each study, a threshold above which an elevated transcriptional activity of MSRV transcripts (High Expression, HE) becomes significant was determined: mean plus two standard deviations of simultaneously tested HBD group. HBD outliers with values beyond HBD mean plus standard deviation, were excluded for normal threshold calculation.

### 2.3 IL6 and CXCL10 in Serum and HSC Cultures

IL6 and CXCL10 protein levels were respectively quantified with Human IL6 ELISA Ready-SET-Go!® and BD OptEIA™ Human IP-10 ELISA Set according to the manufacturer's instructions (eBioscience, Vienna, Austria). The absorbance was read at 450 nm with Biotek EL800 device (Biotek, Luzern, Switzerland).

### 2.4 MSRV-Env Immunohistochemistry in Peripheral Nerve Biopsies

Frozen superficial peroneal nerve biopsies from 7 patients with CIDP and 2 ONDs, one axonal inflammatory neuropathy with perivascular inflammatory changes and one diabetic neuropathy, were collected from the Biological Resource Platform of Henri Mondor University Hospital (to French Ministry of Research #DC-2009-930). Biopsy samples were obtained independently from prospective studies 1 and 2, and used for research purposes according to French regulation (#AC-2014-2056). 15% PFA fixed and paraffin embedded 3 μm-sections were prepared. After paraffin removal, they were washed with Tween-TBS solution. Endogenous peroxidase activity and avidin/biotin proteins were quenched by Biotin blocking system (Dako, Les Ulis, France) according to the manufacturer's instructions. Sections were then washed in Tween-TBS solution, incubated with anti-MSRV-Env mouse monoclonal antibody GN-mAb_04 (Geneuro, Geneva, Switzerland) or an isotype control (Mouse IgG1 kappa, Abcam, Paris, France) diluted at 5 μg/mL in PBS with 10% normal human serum (NHS) for 1 h at room temperature. After washing with Tween-TBS, sections were incubated with a secondary biotinylated antibody (biotin-coupled polyclonal rabbit anti-mouse IgG, Dako, Les Ulis, France) diluted at 10 μg/mL in PBS with 10% NHS for 30 min at room temperature. Sections were washed again in Tween-TBS, streptavidin complex was added for 20 min at room temperature, followed by DAB for 7 min (LSAB + HRP system, Dako, Les Ulis, France). Then, after washing with water and counterstaining with Hemalun, MSRV-Env specific and isotype control staining were examined by light microscopy on two successive sections of each biopsy.

### 2.5 Human Schwann Cells (HSC) Primary Culture, Transfection and Stimulation with MSRV-ENV

HSCs were purchased from ScienCell Research Laboratories (Carlsbad, CA, USA) and cultivated according to the manufacturer's instructions.

#### 2.5.1 Immunocytochemistry

HSCs were grown for 48 h on poly-l-lysine coated (18 h, 37 °C) labtek (Thermo Fisher Scientific, Waltham, MA, USA), fixed in PBS with 4% paraformaldehyde, washed 3 times with PBS and incubated in PBS with 0.01% Triton X-100 for Schwann cell markers (S100β, p75/NGF receptor, P0 myelin protein), or in PBS alone for extracellular TLR4. Nonspecific binding sites were saturated by incubation in PBS with 10% Fetal Bovine Serum for 1 h at 37 °C. They were then incubated with primary antibodies diluted in Fetal Bovine Serum 10% in PBS overnight at 4 °C (rabbit anti-S100, rabbit anti-P0, rabbit anti-P75/NGF at 1/100; Abcam, Paris, France; mouse anti-TLR4 at 1/50; eBioscience, Paris, France). After 3 PBS washes, HSCs were incubated with secondary antibody solutions for 1 h at 37 °C (FITC-coupled goat anti-rabbit IgG diluted 1/400 or FITC-coupled goat anti-mouse IgG diluted 1/200; Millipore, Fontenay-sous-Bois, France). Following 3 PBS washes, plastic chambers were separated from the slides and mounted with Vectashield® mounting medium containing DAPI (Vector Laboratories, Les Ulis, France) before examination by fluorescence microscopy.

#### 2.5.2 Transfections

HSCs were cultured for 24 h in 6-well plates. At 60% confluency, HSC medium was replaced by serum-free OPTIMEM medium (Life Technologies, Saint-Aubin, France). Lipofectamine 2000 was used according to the manufacturer's instructions (Life Technologies, Saint-Aubin, France) to transfect HSC with empty pHBB control plasmid or pHHB plasmids expressing either the full length envelope protein (MSRV-Env-T) or its extracellular domain only (MSRV-Env-SU). After 4 h, transfection medium was replaced by HSC medium and cells were cultured for 48 h with GNbAC1 or GNbAC1 vehicle before collecting culture supernatant and cells for analyses.

#### 2.5.3 HSC Cultures with MSRV-Env

HSCs were grown on 6-wels plates and incubated with endotoxin-free recombinant MSRV-Env or its solubilization buffer (negative control), together with LPS-RS (InVivogen, France), GNbAC1 or GNbAC1 vehicle, at 37 °C for 1 h (IL6) or 4 h (CXCL10) before RNA isolation from cells. Recombinant MSRV-Env was produced in *Escherichia* *coli* and purified as endotoxin-free protein by PX'Therapeutics (Grenoble, France) from plasmid pV14 encompassing the complete env orf cloned from MSRV virion RNA (58 kDa, 542 amino acids, GenBank no. AF331500.1). MSRV Env solubilization buffer (NaCl 150 mM, SDS 1.5%, DTT 10 mM in Trizma–HCL 20 mM, pH 7.5) was provided in parallel.

#### 2.5.4 IL6 and CXCL10 qRT-PCR

After appropriate treatments, HSCs were washed with PBS and total RNA extracted with QIAamp RNeasy Mini Kit. Relative expression of IL6 and CXCL10 to GUS B was performed with Taqman gene expression assays for IL6, CXCL10, and GUS B (Life Technologies, Saint-Aubin, France) according to the manufacturer's instructions.

#### 2.5.5 MSRV-Env ELISA in HSC Cultures

96-well microplates were coated overnight at 4 °C with an anti-MSRV-Env capture antibody (mouse monoclonal GN-mAb_16) diluted at 5 μg/mL in 50 mM bicarbonate buffer, with a 0.05% Tween in PBS, saturated with 1% BSA PBS and washed 4 times. Culture supernatants diluted 1/2 in PBS were then incubated for 2 h at 37 °C, plates washed 4 times and incubated with HRP-coupled anti-MSRV-Env detection antibody (mouse monoclonal GN-mAb_01) for 1 h at 37 °C. After 6 washes, revelation of antigen-bound HRP-antibody used 3,3′,5,5′-tétraméthylbenzidine (30 min reaction, stopped with 2N H2SO4) and absorbance at 450 nm wavelength was measured with Biotek EL800 device (Biotek, Luzern, Switzerland).

### 2.6 Statistical Analysis

Kolmogorov–Smirnov normality test was applied to all data sets. Pearson product moment correlation test, Student's t-test, and one-way analysis of variance followed by Bonferroni's *post hoc* test were used when data passed the normality test, otherwise Spearman rank order correlation test, Mann–Whitney rank sum test, and Kruskal–Wallis one-way analysis of variance on ranks followed by Dunn's *post hoc* test were used. Chi-square or Fisher Exact tests were used to compare rates and proportions. Statistical analyses were performed with SigmaStat 3.5 (Systat inc., San Jose, CA, USA) and data plotted with Prism 5.04 (GraphPad Software, La Jolla, CA).

#### 2.6.1 Funding Sources

The sample collection and the experimental study were financially supported by Geneuro-Innovation, France. The funders had no role in the study design, nor the data collection and analysis, nor in their interpretation or in the writing of the manuscript.

## 3 Results

### 3.1 Demographical and Clinical Characteristics

Demographical and clinical characteristics are presented in Table 1. The male/female ratio in CIDP, OND and HBD groups was not significantly different in Studies 1 or 2. CIDP and OND groups had significantly more males than HBDs in the overall study. CIDP and OND patients were significantly older than HBDs, but CIDP and OND cohorts were matched for age and gender. In CIDP patients, the mean disease duration was 7.2 ± 1.1 years, ranging from 9 weeks to 47 years. They were treated by IVIG (47%), oral immunosuppressant (16%), different regimen with corticosteroids and 27% were untreated at inclusion (Table 1).

**Table 1:** Demographic characteristics by study and type of biological analyses.

		Group			P value		
		HBD	CIDP	OND	HBD/CIDP	HBD/OND	CIDP/OND
Study 1	Gender PCR MSRV env n male/n female	10/10	11/4	–	0.296	–	–
	Age PCR MSRV env median (range)^a^	43,5 (37–51)	59 (28–89)	–	**0.040**	–	–
	Gender PCR MSRV-pol n male/n female	8/9	11/4	–	0.250	–	–
	Age PCR MSRV-pol median (range)^a^	44 (37–51)	59 (28–89)	–	0.056	–	–
Study 2	Gender PCR MSRV env n male/n female	14/14	10/7	9/3	0.789	0.264	0.449
	Age PCR MSRV env median (range)^a^	42 (22–63)	57 (36–79)	53 (43–82)	**< 0.001**	**< 0.001**	1.000
	Gender PCR MSRV-pol n male/n female	12/14	8/6	5/1	0.740	0.178	0.354
	Age PCR MSRV-pol median (range)^a^	42 (22–63)	57.5 (36–79)	65 (49–82)	**< 0.001**	**< 0.001**	0.794
Studies 1 + 2	Gender IL-6 serum n male/n female	28/34	35/12	16/3	**0.004**	**0.006**	0.524
	Age IL-6 serum median (range)^a^	42.5 (22–69)	60 (28–89)	53 (43–82)	**< 0.001**	**< 0.001**	1.000
	Gender CXCL10 serum n male/n female	31/34	35/14	16/3	**0.019**	**0.008**	0.359
	Age CXCL10 serum median (range)^a^	43 (22–69)	60 (28–89)	53 (43–82)	**< 0.001**	**< 0.001**	1.000
	Treatment (% of CIDP patients)						
	azathioprine		8%				
	cyclosporine		8%				
	IVIG^b^		49%				
	methylprednisolone		2%				
	none		27%				
	plasmapheresis		2%				
	prednisone		2%				
	prednisone + tacrolimus		2%				

In bold: statistically significant differences

a Years.

b Intravenous human immunoglobulins.

### 3.2 Elevated MSRV Transcripts in PBMC from CIDP.

**Fig. 1:**
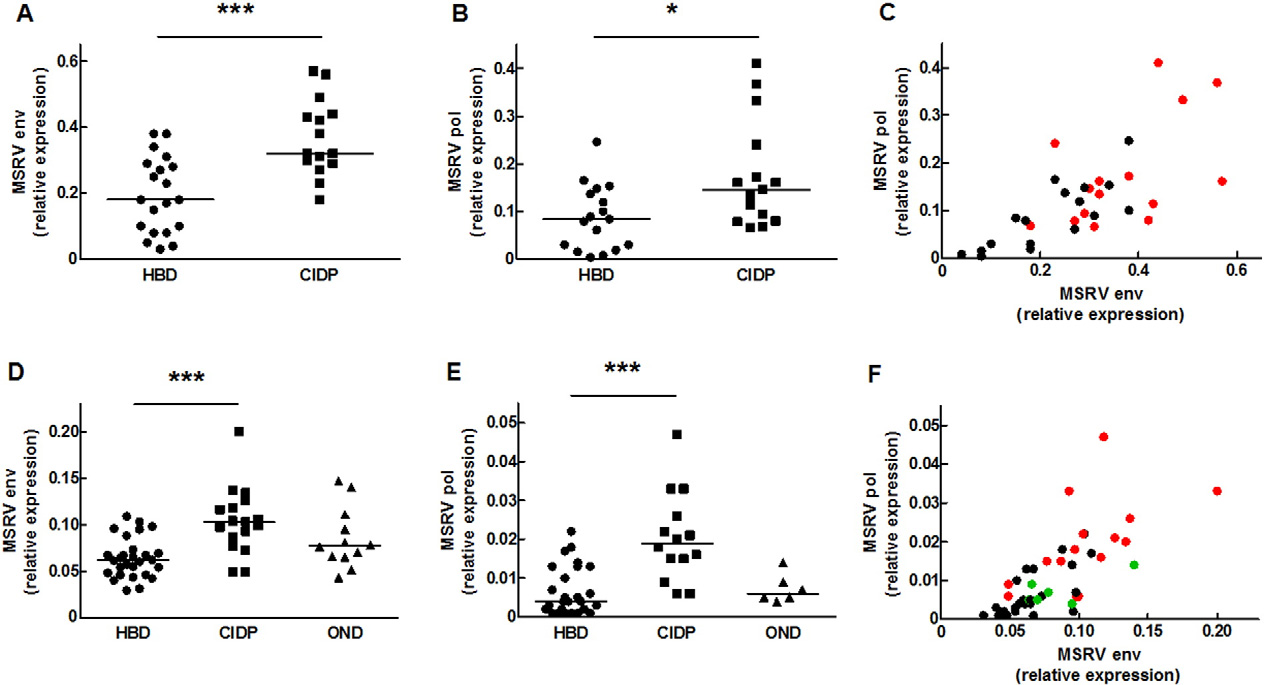
MSRV-Env and MSRV-Pol transcripts levels are increased in peripheral blood mononuclear cells of CIDP patients in two independent studies. MSRV-Env (Study 1: A; Study 2: D) and MSRV-Pol (Study 1: B; Study 2: E) RNA levels were quantified by qRT-PCR in PBMC from healthy blood donors (HBDS), CIDP patients (CIDP), and other neurological diseases controls (ONDs). MSRV-Env and MSRV-Pol expressions are strongly correlated in HBDS (black dots), OND (green dots) and CIDP (red dots) in Studies 1 and 2 (respectively C and F). Data are expressed as relative expression of the targeted transcript to GUS B reference transcript, and plots represent individual values. *P < 0.05, ***P < 0.001 versus HBDS.

Results are illustrated in Fig. 1 and statistic analyses are presented in Table 2.

**Table 2:** MSRV transcripts, IL6 and CXCL10 expressions in CIDP peripheral blood mononuclear cells and serum compared to OND and normal populations.

		Group			P value			Correlation
		HBDS	CIDP	OND	HBDS/CIDP	HBDS/OND	CIDP/OND	r; p value
Study 1	MSRV env RNA mean ± SEM (n)^1^	0.19 ± 0.03 (20)	0.37 ± 0.03 (15)	–	**< 0.001**	–	–	} 0.713; **< 0.001**
	MSRV-pol RNA mean ± SEM (n)^1^	0.09 ± 0.02 (17)	0.17 ± 0.03 (15)	–	**0.011**	–	–	
	MSRV env HE subjects ratio (%)^2^	0/20 (0%)	6/15 (40%)	–	**0.003**	–	–	
	MSRV-pol HE subjects ratio (%)^2^	1/17 (6%)	4/15 (27%)	–	0.161	–	–	
	MSRV-env and pol HE subjects ratio (%)^2^	0/17 (0%)	3/15 (20%)	–	0.092	–	–	
	MSRV-env or -pol HE subjects ratio (%)^2^	1/17 (6%)	7/15 (47%)	–	**0.013**	–	–	
Study 2	MSRV env RNA mean ± SEM (n)^1^	0.064 ± 0.004 (28)	0.104 ± 0.009 (17)	0.085 ± 0.009 (12)	**< 0.001**	0.114	0.538	} 0.782; **< 0.001**
	MSRV-pol RNA mean ± SEM (n)^1^	0.007 ± 0.001 (26)	0.021 ± 0.003 (14)	0.007 ± 0.002 (6)	**< 0.001**	1.000	**0.067**	
	MSRV env HE subjects ratio (%)^2^	2/28 (7%)	9/17 (53%)	3/12 (25%)	**< 0.001**	0.149	0.251	
	MSRV-pol HE subjects ratio (%)^2^	3/26 (12%)	8/14 (57%)	0/6 (0%)	**0.007**	1.000	**0.042**	
	MSRV-env and -pol HE subjects ratio (%)^2^	2/26 (8%)	6/14 (43%)	0/6 (0%)	**0.014**	1.000	0.115	
	MSRV-env or -pol HE subjects ratio (%)^2^	3/26 (12%)	9/14 (64%)	1/6 (17%)	**< 0.001**	1.000	**0.050**	
	MSRV env RNA/CIDP disease duration^3^							− 0.580; **0.014**
	MSRV-pol RNA/CIDP disease duration^3^							− 0.643; **0.012**
Studies 1 + 2	IL-6 detected in serum ratio (%) range^4^	5/62 (8%) 3–14	14/47 (30%) 3–387	2/19 (11%) 4–10	**0.007**	0.664	**0.007**	
	CXCL10 in serum mean ± SEM (n)^4^	81 ± 7 (65)	115 ± 10 (49)	60 ± 13 (19)	**0.007**	0.133	**< 0.001**	

In bold: statistically significant differences

1 Relative expression of the targeted RNA to GUS B RNA.

2 HE: High Expression of the targeted RNA.

3 Years.

4 IL-6 and CXCL10 concentrations in serum expressed in pg mL^− 1^.

In Study 1, PBMC mRNA from 20 HBD and 15 CIDP patients passed quality criteria (Cf. Methods). MSRV-env (p < 0.001; Fig. 1A) and -pol (p < 0.05; Fig. 1B) expressions were significantly higher in CIDP patients than in HBDs. MSRV-env expression was significantly correlated to MSRV-pol expression (r = 0.713; p < 0.001), and highest MSRV-env & -pol dual expressions were found in CIDP patients (Table 2; Fig. 1C).

In Study 2, PBMC mRNA passed quality criteria in 28 HBD, 17 CIDP and 12 OND samples for MSRV-env and in 26 HBD, 14 CIDP and 6 OND samples for MSRV-pol. Results confirmed that MSRV-env and -pol expressions were elevated in CIDP patients, but not in OND, when compared to HBD group (Fig. 1D and E respectively; p < 0.001 for both transcripts). MSRV-env expression was again correlated to MSRV-pol expression (r = 0.782; p < 0.001) with highest dual MSRV-env and -pol expressions in CIDP (Table 2; Fig. 1F).

A threshold above which mRNA levels were significantly elevated (High Expression; HE) was calculated (Cf. Methods). In Study 1, 6 CIDP patients had HE for MSRV-env (40%) but none in HBDs (0%; p < 0.01). 4 s (27%) and 1 HBD (6%) had HE for MSRV-pol (p = 0.161). 7/15 CIDPs (47%; p < 0.05 versus HBDs) had HE for at least one MSRV transcript. A dual MSRV env/pol HE profile was observed in 0/17 HBDs and 3/15 CIDPs (20%, p = 0.092).

In Study 2, 2 HBDs (7%) versus 6 CIDP (53%) presented HE for MSRV-env (p < 0.001). 3 ONDs (25%) had MSRV-env HE, which was not significantly different from HBD or CIDP groups. 8 CIDP (57%) had HE for MSRV-pol, which was significantly different from both HBD (12%; p < 0.01) and OND (0%; p < 0.05) groups. 9/14 CIDP (64%; p < 0.001 and p = 0.05 versus HBDs and ONDs respectively) presented a HE profile for at least one MSRV transcript. A dual HE profile was observed in 2/26 HBDs (8%), in 0/6 OND and in 6/14 CIDPs (43%; p < 0.01 versus ONDs or HBDs; Table 2).

No correlation existed between MSRV-env or -pol expressions and the age of subjects, the age at disease onset, the gender or the treatment. Though not evidenced in Study 1, MSRV-env (r = − 0.580; p < 0.05) and -pol (r = − 0.643; p < 0.05) expressions in CIDP patients are inversely correlated to the disease duration in Study 2 (Table 2).

### 3.3 Elevated IL6 and CXCL10 Levels in CIDP Serum

We analyzed IL6 cytokine and CXCL10 chemokine levels in sera from Studies 1 and 2. Statistics of comparisons between groups are presented in Table 2.

IL6 values above the limit of detection were more frequent in CIDP (30%) than in ONDs (11%) and HBDs (8%), which was statistically significant (p < 0.01). 2 CIDP patients presented highly elevated IL6 (230 and 387 pg/mL) whereas the highest IL6 levels in ONDs and HBDS were 10 and 14 pg/mL respectively. Similarly, CXCL10 levels were significantly higher in serum of CIDP patients (115 ± 10 pg/mL) than in HBDS (81 ± 7 pg/mL; p < 0.01) and ONDs (60 ± 13 pg/mL; p < 0.001). No correlation existed between IL6 and CXCL10 expressions, nor with the age of subjects, the age at disease onset, the gender, the treatment regimen, nor even with MSRV-env and -pol mRNA levels.

### 3.4 MSRV-Env Expression in Nerve Biopsies of Patients with CIDP

**Fig. 2:**
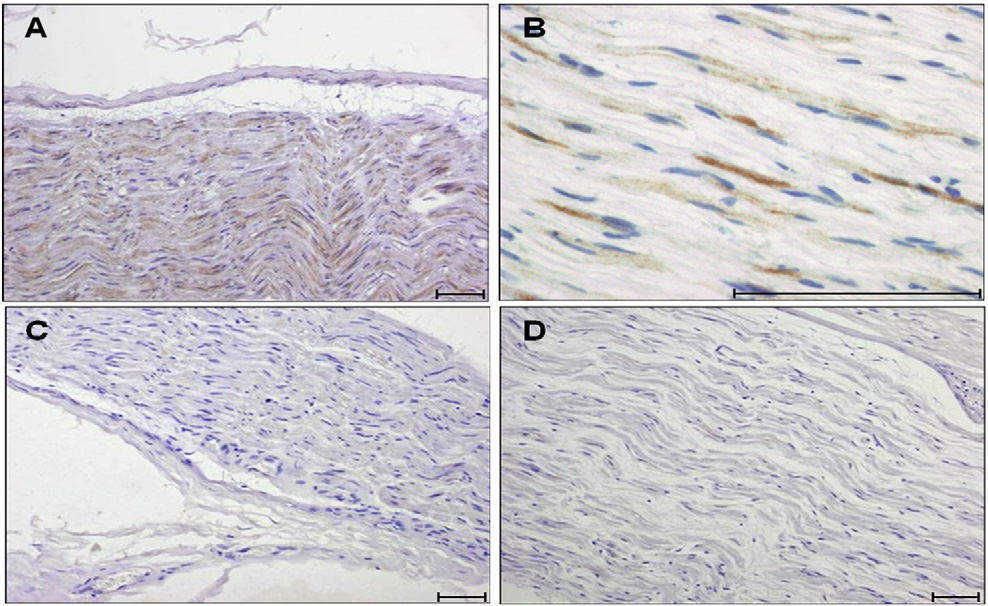
MSRV-Env is expressed in peripheral nerves biopsies from CIDP patients. Representative immunohistological analysis showing that MSRV-Env immunoreactivity (brown) is found in the cytoplasm of Schwann cells (low magnification: A; high magnification: B). No staining is observed in the corresponding serial section of the same biopsy incubated with a non-relevant isotype antibody (C) or in a biopsy from a control neuropathy (D). Scale bar: 0.5 μm.

Distal sensory peripheral nerve (PN) biopsies were examined with an MSRV-Env specific monoclonal antibody or an isotype control antibody. MSRV-Env was detected in biopsies from five CIDP patients out of seven tested (71%), in the absence of staining with the isotype control. As shown in Fig. 2, MSRV-Env staining was localized in the cytoplasm of Schwann cells (SC) (4/5) or in the myelin sheath (1/5). MSRV-Env staining was not seen in the two control biopsies, one of which showed inflammatory infiltrates.

### 3.5 HSC Expressing MSRV-Env Protein Produce and Release IL6 and CXCL10

**Fig. 3:**
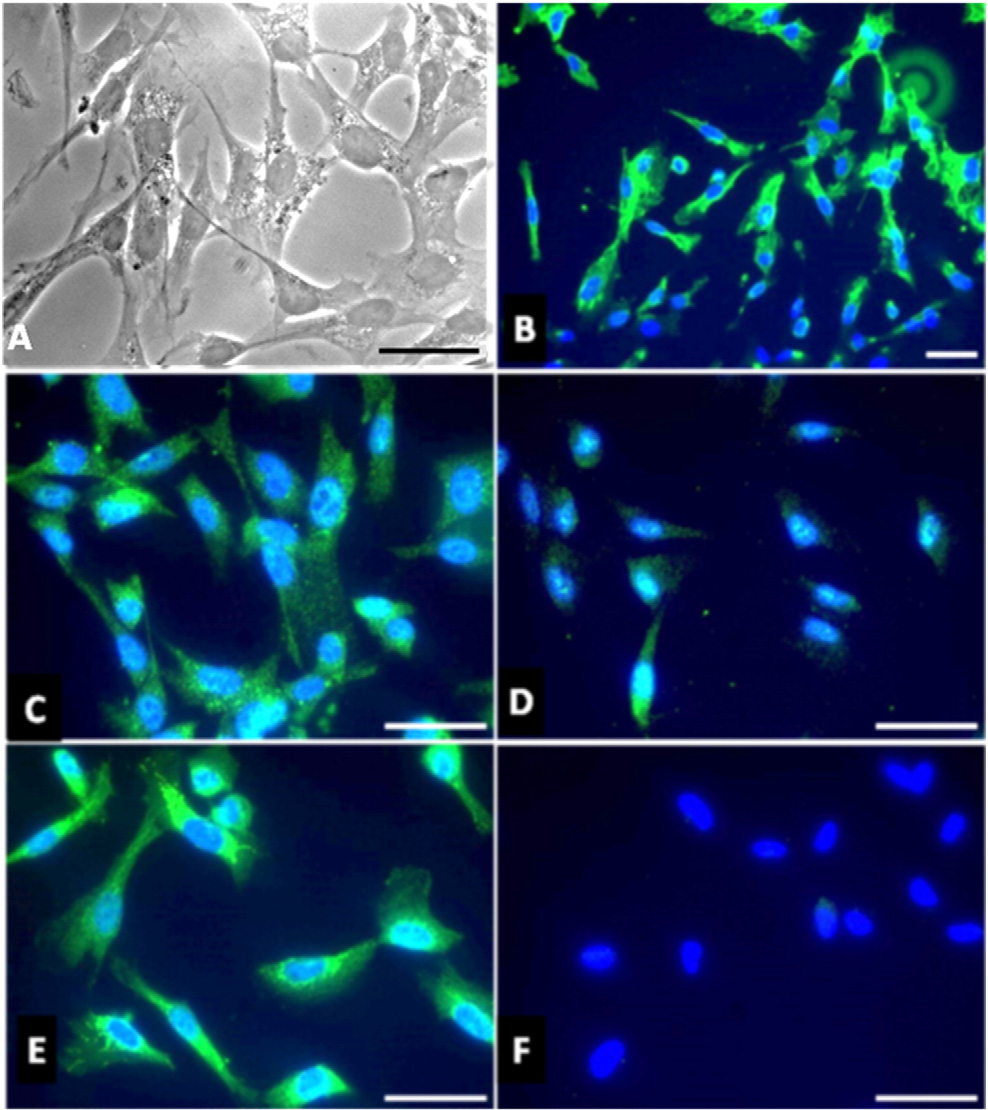
Characterization of human Schwann cells in primary culture. Morphology of HSC in light microscopy (A). Immunocytofluorescence analyses show that HSC in primary culture strongly express TLR4 (B), S100β (C), P0 myelin protein (D), and P75/NGF receptor (E). No staining is observed when the secondary antibody coupled to FITC is used alone (F). Cell nuclei are labelled with DAPI (blue), scale bar: 5 μm.

We then studied the effects of MSRV-Env expression on in vitro transfected HSC, mimicking MSRV-Env expression in HSC as observed in CIDP nerve biopsies. Primary cultured HSCs were transfected with plasmids encoding the complete MSRV-env protein (Env-T) or encoding its extracellular domain (Env-SU). Immunocytofluorescence analyses showed that HSCs strongly express toll-like receptor 4 (TLR4), the pharmacological target of MSRV-Env, at their surface (Madeira et al., 2016). HSC can thus potentially respond to MSRV-Env stimulation. HSC phenotype was confirmed by typical morphology with concomitantly strong expression of the calcium binding S100β protein, P0 myelin protein, and P75/NGF receptor. Specific detection was confirmed by the absence of staining when incubation only used the secondary fluorescent antibody (Fig. 3).

**Fig. 4:**
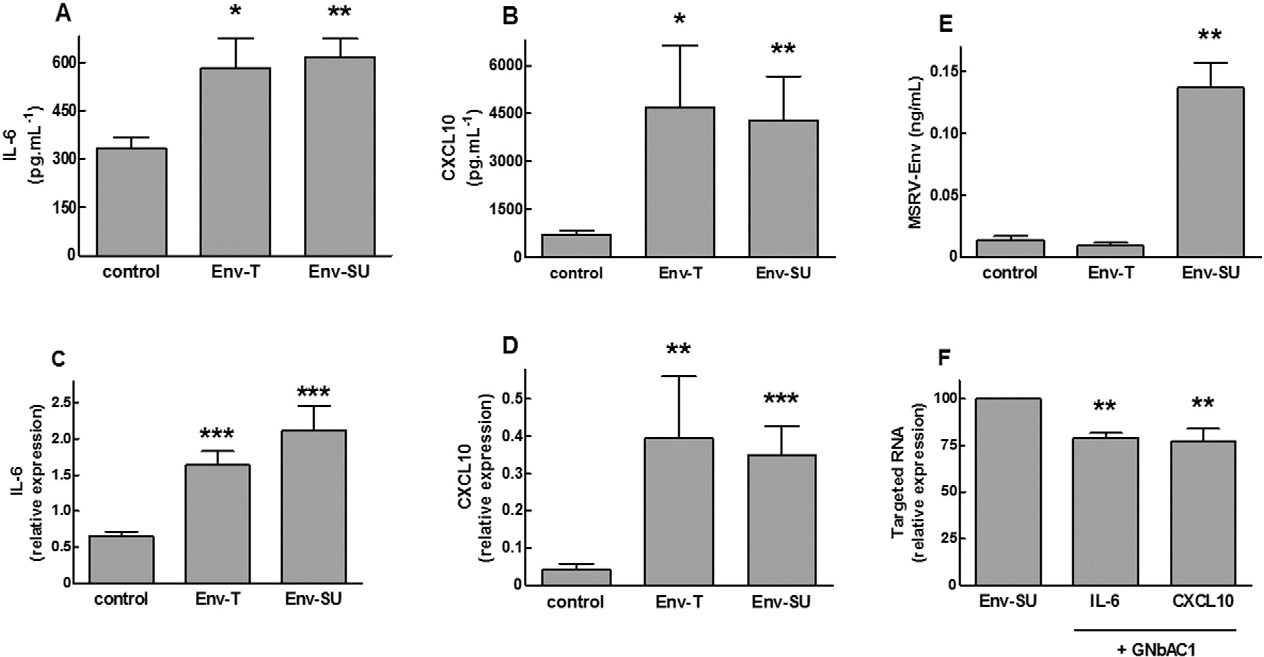
Human Schwann cells expressing MSRV-Env produce and release IL6 and CXCL10. HSCs were transfected with a plasmid encoding the full length MSRV-Env (Env-T) or a fraction of the extracellular domain of MSRV-Env (Env-SU), or the corresponding empty plasmid (control). Culture media and HSC transcripts were isolated 48 h after transfection. IL6 (A; F) and CXCL10 (B; F) transcripts levels were quantified by qRT-PCR. IL6 (C), CXCL10 (D) and MSRV-Env-SU (E) proteins levels in the culture media were quantified by ELISA. HSCs were incubated without (Env-SU) or with GNbAC1 (+ GNbAC1) at 200 nM for 48 h after transfection with MSRV-Env-SU (F). Data are expressed in pg/mL (A; B), ng/mL (E), or as relative expression of the targeted transcript to GUS B reference transcript (C; D; F) and represent Mean ± SEM of 6 to 9 experiments. *P < 0.05, **P < 0.01, ***P < 0.001 versus control (A–E) or MSRV-Env-SU (F).

As presented in Fig. 4, HSC expressing MSRV-Env or MSRV-Env-SU for 48 h presented a strong and significant increase of IL6 and CXCL10 transcripts levels (respectively, + 151% with p < 0.001 and + 887% with p < 0.01, for Env-T, as well as, + 226%and + 777% both with p < 0.001, for Env-SU). MSRV-Env also induced an important release of IL6 and CXCL10 proteins (respectively, + 75% and + 555% both with p < 0.05 for Env-T as well as, + 85% and + 499% both with p < 0.01 for Env-SU; Fig. 4A–D). Dosage of MSRV-Env protein in culture media of transfected HSC showed that Env-T was mostly sequestered at the plasma membrane level and that a MSRV-Env-SU was better released from HSC. The specificity of this MSRV-Env mediated effect was shown with a highly specific neutralizing antibody (GNbAC1, 200 nM) added shortly after HSC transfection, which significantly inhibited the increase of IL6 (− 21%; p < 0.01) and CXCL10 (− 23%; p < 0.01) transcripts induced by MSRV-env-SU (Fig. 4E–F).

### 3.6 GNbAC1 Inhibits MSRV-Env Induced IL6 and CXCL10 in HSC

**Fig. 5:**
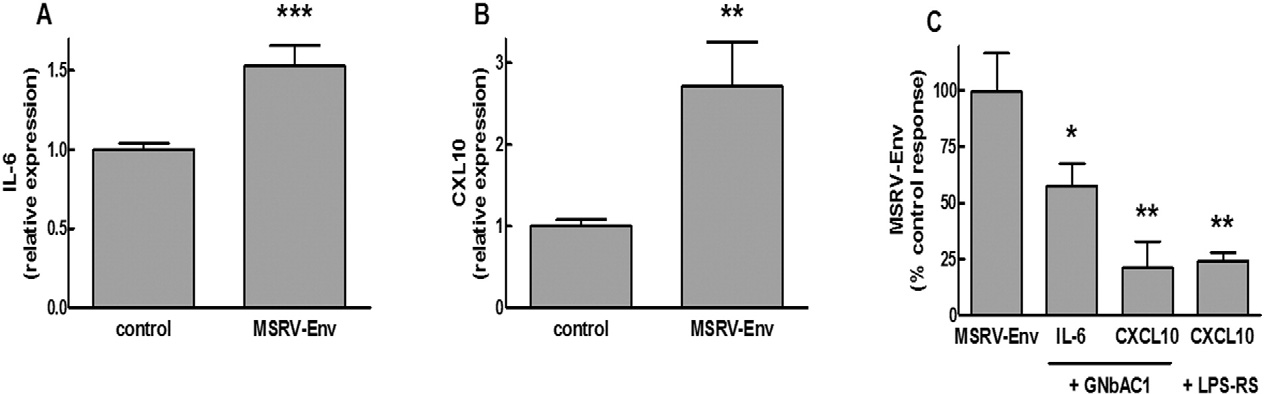
GNbAC1 inhibits IL6 and CXCL10 expressions induced by MSRV-Env in human Schwann cells. HSCs were stimulated 1 h (IL6) or 4 h (CXCL10) with recombinant full length MSRV-Env (3 nM) alone or together with GNbAC1 (200 nM) or LPS-RS (300 ng mL^− 1^) before RNA isolation. IL6 (A; C) and CXCL10 (B; C) transcripts levels were quantified by qRT-PCR. Data are expressed as relative expression of the targeted transcript to GUS B reference transcript (A; B) or as % of control response to MSRV-Env (in the absence of GNbAC1 or LPS-RS) and represent Mean ± SEM of 6 to 8 experiments. *P < 0.05, **P < 0.01, ***P < 0.001 versus control (A; B) or MSRV-Env (C).

We developed experimental conditions appropriate for a pharmacological evaluation of MSRV-Env and GNbAC1 by stimulating HSC for 4 h with purified and endotoxin-free recombinant MSRV-Env protein before HSC transcript isolation. A low concentration of MSRV-Env (3 nM) induced a significant increase of IL6 (+ 53%; p < 0.001) and CXCL10 (+ 172%; p < 0.01) transcript levels in HSC (Fig. 5A–B). The increase of IL6 and CXCL10 expressions induced by MSRV-Env in HSC was significantly inhibited by 200 nM GNbAC1 (− 43%; p < 0.05 and − 79%; p < 0.01 respectively) and by LPS-RS, a competitive TLR4 antagonist (Stevens et al., 2013) (− 76%; p < 0.01; Fig. 5C).

## 4 Discussion

After initial detection of HERV-W protein, MSRV-Env, in CIDP cases within a control group of inflammatory neurological diseases for a study on MS (Perron et al., 2012), the present study confirms an association between MSRV-Env expression and CIDP. This is now supported by significantly upregulated MSRV-env mRNA transcription in PBMC, by MSRV-Env protein detection in CIDP peripheral nerve lesions with dominant expression in Schwan cells and, with evidence of its target receptor TLR4 on HSC, by direct pro-inflammatory effects of MSRV-Env iducing IL6 and CXCL10 release from HSC. Overall, about half of the CIDP patients showed a High Expression profile for MSRV env or pol. But considering subjects presenting a HE profile for at least one MSRV transcript, up to 65% were found in CIDP population when only 12% in HBDs and 17% in ONDs. Since env- and pol-encoded proteins are expressed from different mRNAs (Blond et al., 1999), their correlated upregulation in CIDPs also indicates that MSRV genome and/or HERV-W related elements are globally upregulated but also confirm the specificity of coincident results with two different HERV-W genes.

MSRV-Env has for long been shown to induce innate immune dysregulation, autoimmunity and inflammation in cellular and animal models (Perron et al., 2001, Rolland et al., 2006). TLR4 activation revealed to be a prerequisite for the activation of cytopathogenic pathways and physiopathological cascades by this endogenous protein and, as a consequence of its expression, pro-inflammatory cytokines and chemokines have regularly been evidenced (Rolland et al., 2006, Kremer et al., 2013, Madeira et al., 2016, Duperray et al., 2015). In the present study two relevant ones for CIDP and Schwann cells, IL-6 and CXCL10, were studied.

Unlike available previous studies having investigated IL6 in sera of CIDP patients (Maimone et al., 1993, Sainaghi et al., 2010), we found elevated IL6 and CXCL10 levels in CIDP serum when compared to HBDs, without confounding correlation with age and gender. These diverging findings can easily be understood when considering that our study showed detectable levels of IL6 in about 30% of 47 CIDP sera, whereas these previous works having tested 7 or 8 patients only had the greatest probability not to include a single case with detectable level. CXCL10, a chemokine known as a chemoattractant for macrophages and T cells (Xuan et al., 2014) was previously detected in the CSF of CIDP patients in correlation to the degree of inflammation in proximal segments of spinal nerve roots and to blood nerve barrier damages (Kieseier et al., 2002, Mahad et al., 2002), and was also detected in CIDP lesions (Kieseier et al., 2002). Similarly, IL6 was detected in the CSF (Maimone et al., 1993) and in sural nerve biopsies of CIDP patients (Lindenlaub and Sommer, 2003, Yamamoto et al., 2002). Additionally, Schwann cells can produce CXCL10 (Medeiros et al., 2015), as well as IL6 (Lu et al., 2009), most particularly after exposure to LPS, a TLR4 agonist like MSRV-Env (de Leseleuc et al., 2013). The present study with HSC cultures shown to be TLR4-positive, exposed to or expressing MSRV-Env, demonstrated a potent induction of both IL6 and CXCL10. As in previous studies on MSRV-Env pathogenic mode of action (Rolland et al., 2006, Kremer et al., 2013, Madeira et al., 2016, Duperray et al., 2015), a specific TLR4-driven effect was also confirmed in HSC with the observed inhibition of MSRV-Env effects by LPS-RS, a competitive TLR4 antagonist. Thus, MSRV-Env can directly trigger HSC to release pro-inflammatory effectors through TLR4 activation and signaling pathways.

It therefore appears that an autoimmune reaction in CIDP may result from a TLR4-driven activation of innate immunity by MSRV-Env protein in immune and neuroglial cell types with potential downstream superantigen-like effects when T-cells are recruited and exposed to MSRV-Env (Perron et al., 2001). A mechanism of TLR-conditional activation of lymphocyte by innate immune and/or antigen presenting cells has now been evidenced (Kool et al., 2011), which may differentiate eventual TLR-dependent superantigen effects from the direct T-cell polyclonal activation observed with bacterial superantigens (Muller-Alouf et al., 2001). In all instances, superantigenic effects cause antigen-independent polyclonal activation of T lymphocytes, which was incriminated in MS (Rudge, 1991) and was more recently experimentally evidenced with MSRV-Env induction of autoimmunity against the central nervous system myelin proteins in animal models (Perron et al., 2013). Consistent with this known pathogenic potential, MSRV-Env protein expression as observed in HSC within CIDP peripheral nerve lesions may therefore trigger inflammation along peripheral nerves mirrored by systemic immune dysregulation. Of note, MSRV-Env was not detected in similar biopsy from a control case presenting inflammatory lesions with perivascular leukocyte infiltration, which adds to the demonstration that MSRV-Env is not a consequence of inflammation, but the reverse.

Thus, MSRV-Env cannot simply represent a peripheral biomarker. Nonetheless, as MSRV-env and MSRV-pol expressions were inversely correlated to disease duration, MSRV activity may peak at early stages of the disease and its early quantification could be of value for CIDP phenotyping or diagnosis. As it may also reflect some efficacy of long-term treatments on its recirculation or expression in the bloodstream, longitudinal and/or transversal studies of accurately representative cohorts should now be envisaged.

Finally, the present study also showed that the strong pro-inflammatory upregulation of IL6 and CXCL10 induced by MSRV-Env in HSC was significantly inhibited by a specific neutralizing antibody targeting MSRV-Env, GNbAC1. As this antibody is a humanized therapeutic IgG4, now in phase II clinical trials in MS (Curtin et al., 2015, Derfuss et al., 2015, Zimmermann et al., 2015), this indicates potential new avenues for the treatment of CIDP patients with significantly elevated MSRV expression.

Concluding from these original findings, we propose that MSRV-Env is a potential therapeutic target in CIDP, at least in a significant proportion of patients, and that it may become a useful blood biomarker along with CXCL10 and IL6. This also provides arguments in favor of the humanized monoclonal antibody GNbAC1, as a potentially innovative treatment to be evaluated in CIDP.

## Potential Conflicts of Interest

RF, AM, NG, IB, MB, CB, FC, ABL, and HP are employees of GeNeuro Innovation SAS or GeNeuro SA.

AJS and TK perform consultancy work for Actelion Pharmaceuticals Switzerland.

AC gave expert testimony for CSL Behring, Novartis, received grants from Biogen, Novartis, CSL Behring, Geneuro, Octapharma, and gave lectures for Genzyme.

## Authors' Contributions

RF, AM: developed, supervised, and interpreted all protocols and experiments, except clinical and sample recruitment, interpretation of clinical data and immunohistological analysis of biopsies. They contributed to the text of the manuscript and to its successive reviews.

NG, IB, MB: developed and performed all experiments except biopsies analyses, reviewed the manuscript.

JA: biopsies analyses, reviewed the manuscript.

FC, ABL, AJS, interpreted data and reviewed the manuscript.

PAP, CL, CB, TK, AC: set-up of the clinical studies, patients' recruitment, data interpretation, reviewed the manuscript.

HP, AC, FC and TK initiated and supervised the study.

HP: reviewed contributions, corrected and finalized the text of manuscript as well as that of the revised version.

## Appendix A Supplementary data

Supplementary material Image 1
